# Supplementary material for: HoxC5 and miR-615-3p target newly evolved genomic regions to repress hTERT and inhibit tumorigenesis
Source: Nat Commun. 2018 Jan 8;9:100. doi: 10.1038/s41467-017-02601-1 (PMC5758779; doi:10.1038/s41467-017-02601-1)
Supplement: Supplementary file 1 — supplementary info [file 41467_2017_2601_MOESM1_ESM.pdf]

Supplementary Figures

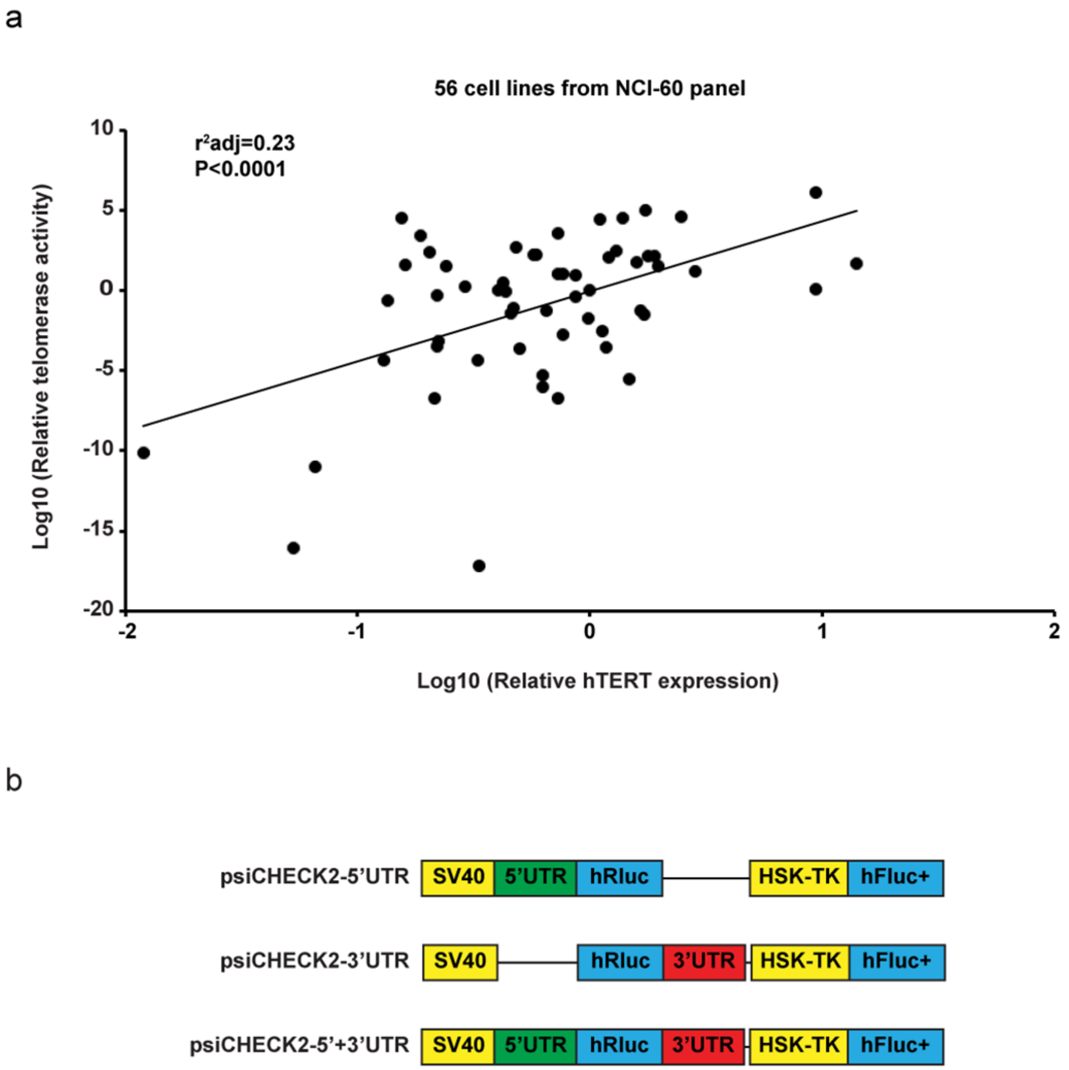

Supplementary Figure 1: The expression level of *hTERT* mRNA correlates with telomerase activity in cancer cells.

a. Scatter plot for relative telomerase activity and *hTERT* mRNA expression in 56 human cancer cell lines in NCI-60 panel.

b. Schematic representation of the psiCHECK2-3'UTR, psiCHECK2-5'UTR and psiCHECK2-5'+3'UTR reporter constructs.

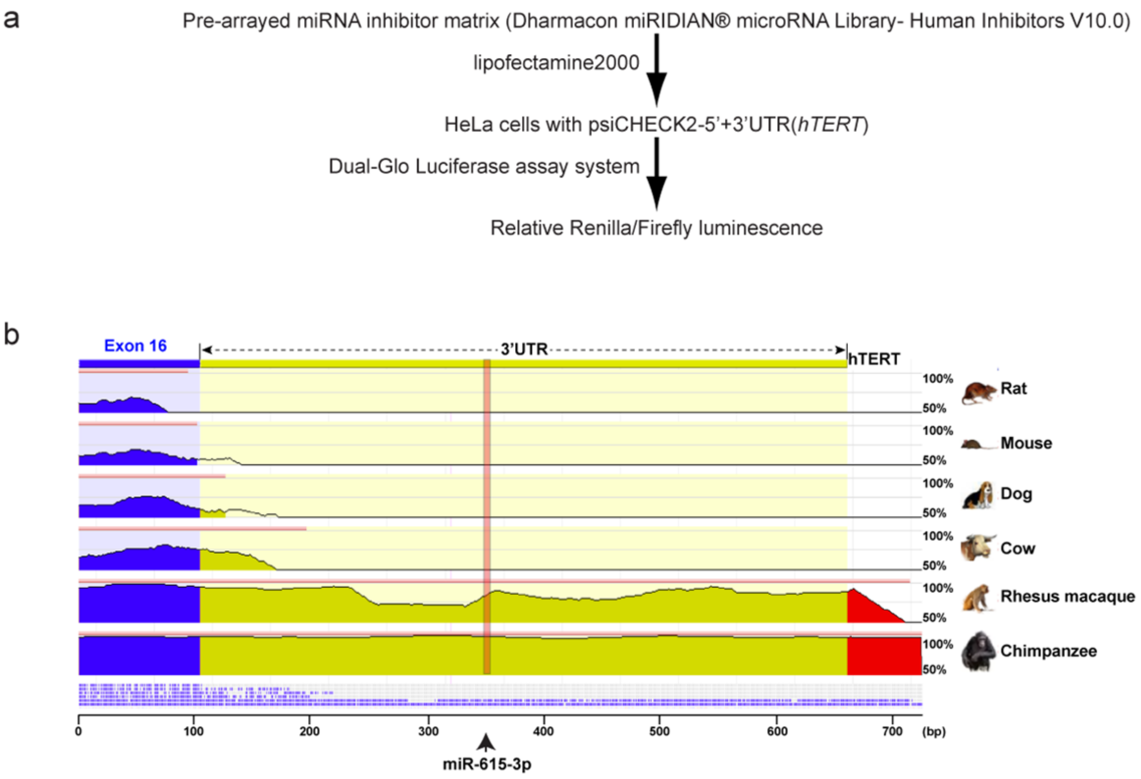

Supplementary Figure 2: Genome-wide miRNA inhibitor screen identify miR-615-3p targeting *hTERT* 3'UTR.

a. Design of the genome-wide miRNA inhibitor library screen to identify miRNAs that target *hTERT* UTRs.

b. The 3′UTR sequences of the *TERT* mRNA from rat, mouse, dog, cow, rhesus macaque, chimpanzee and human were compared using the ECR-Browser software (http://ecrbrowser.dcode.org). The height of the curves (50% < X < 100%) indicates the homology (blue = exon, yellow = untranslated regions of the RNA, red = intergenic regions, pink line above indicate evolutionary conserved region). The miR-615-3p target site is indicated by arrowhead and highlighted in pink.

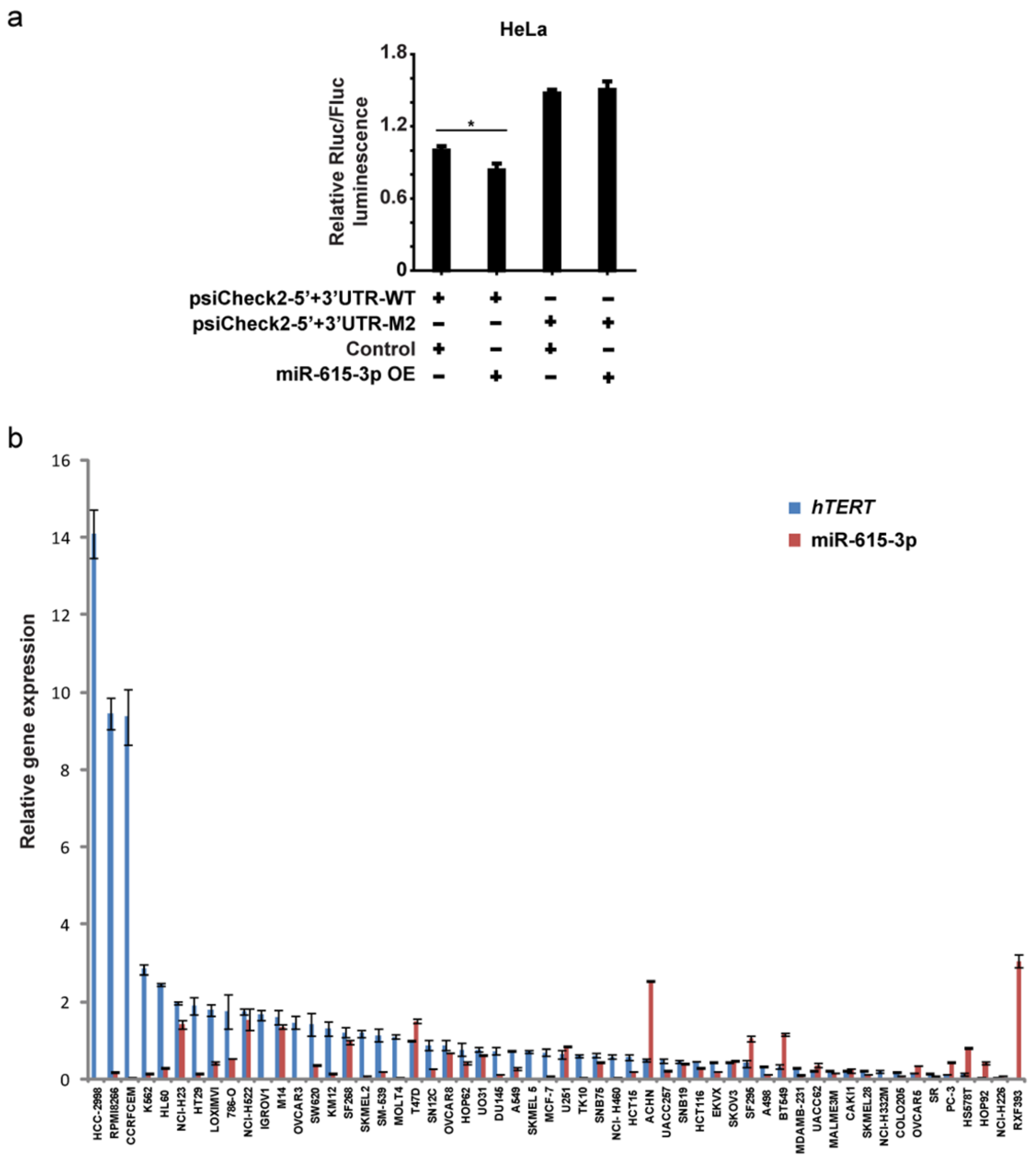

Supplementary Figure 3: Regulation of *hTERT* expression by miR-615-3p.

a. miR-615-3p targets *hTERT* 3'UTR to suppress *hTERT* expression. Significance was determined by t-test. * $P \leq 0.05$.

b. The relative expression of *hTERT* mRNA and miR-615-3p in 56 human cancer cell lines in NCI-60 panel as quantified by real-time RT-PCR. The expression of *hTERT* mRNA and miR-615-3p in 293T cells was set as 1.

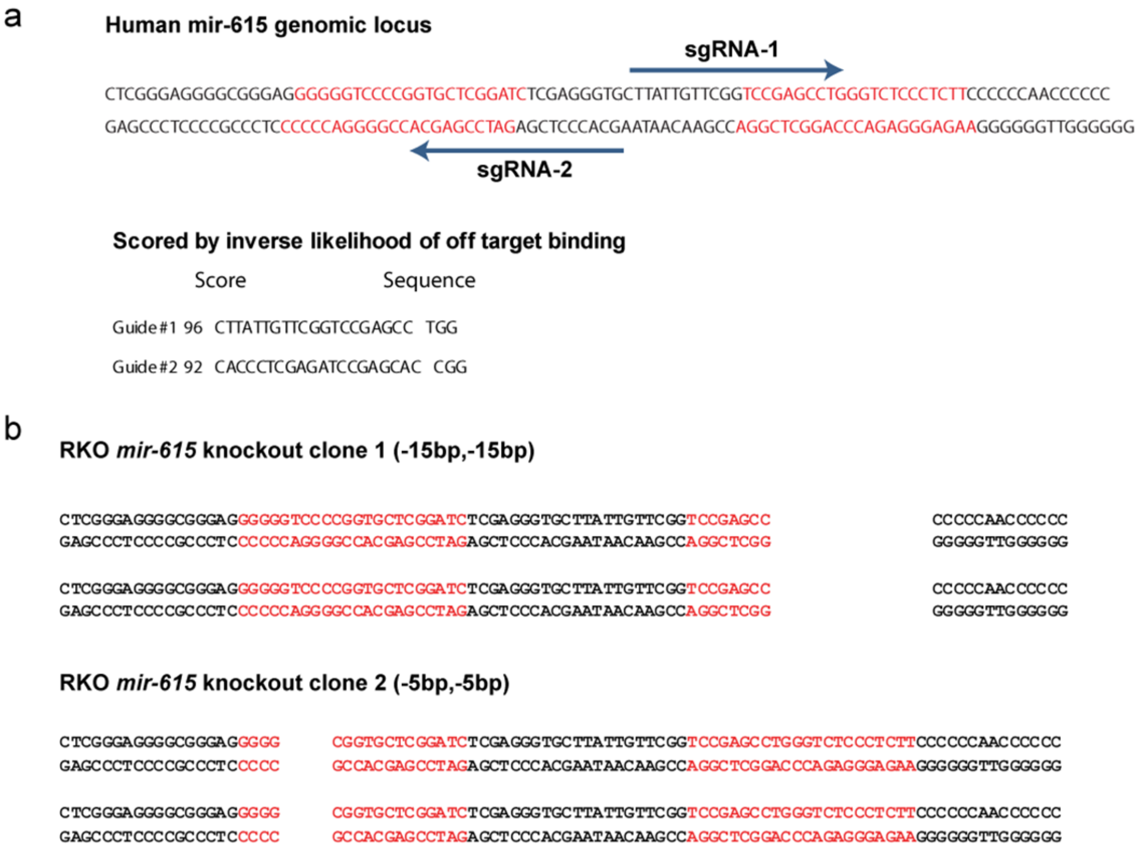

Supplementary Figure 4: CRISPR/Cas9-mediate knockout of *mir-615-3p* in RKO cells.
a. Schematic design of CRISPR sgRNAs for knockout of *mir-615-3p*.
b. Sanger sequencing confirmed the small deletions in *mir-615* hairpin region, which is crucial for the maturation of miR-615-3p in two independent clonal derived RKO cell lines.

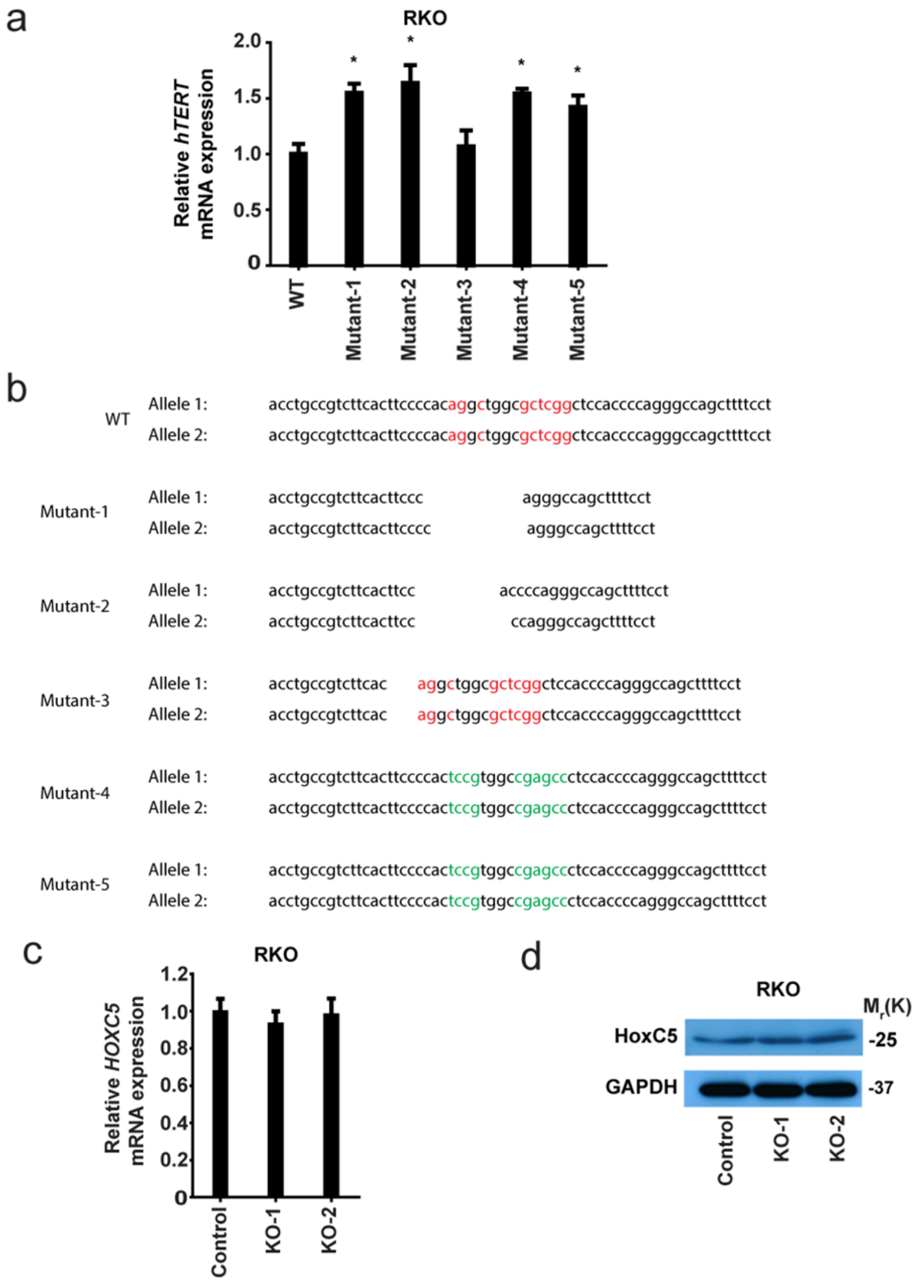

Supplementary Figure 5: Mutation of miR-615-3p binding site in the endogenous *hTERT* 3'UTR resulted in increased *hTERT* mRNA expression.

a. Relative *hTERT* mRNA expression in parental RKO cells or clonal derived RKO cell lines with small deletions or mutations in the *hTERT* 3'UTR. Significance was determined by t-test. * $P \leq 0.05$.

b. Sanger sequencing confirmed the small deletions and mutations in *hTERT* 3'UTR region. The seed region and the additional conserved site are highlighted in red. The mutated bases are highlighted in green.

c. Real-time RT-PCR showing the expression of endogenous *HOXC5* mRNA in parental and *mir-615-3p* knockout RKO cell lines.

d. Western blotting showing the expression of endogenous HoxC5 in parental and *mir-615-3p* knockout RKO cell lines.

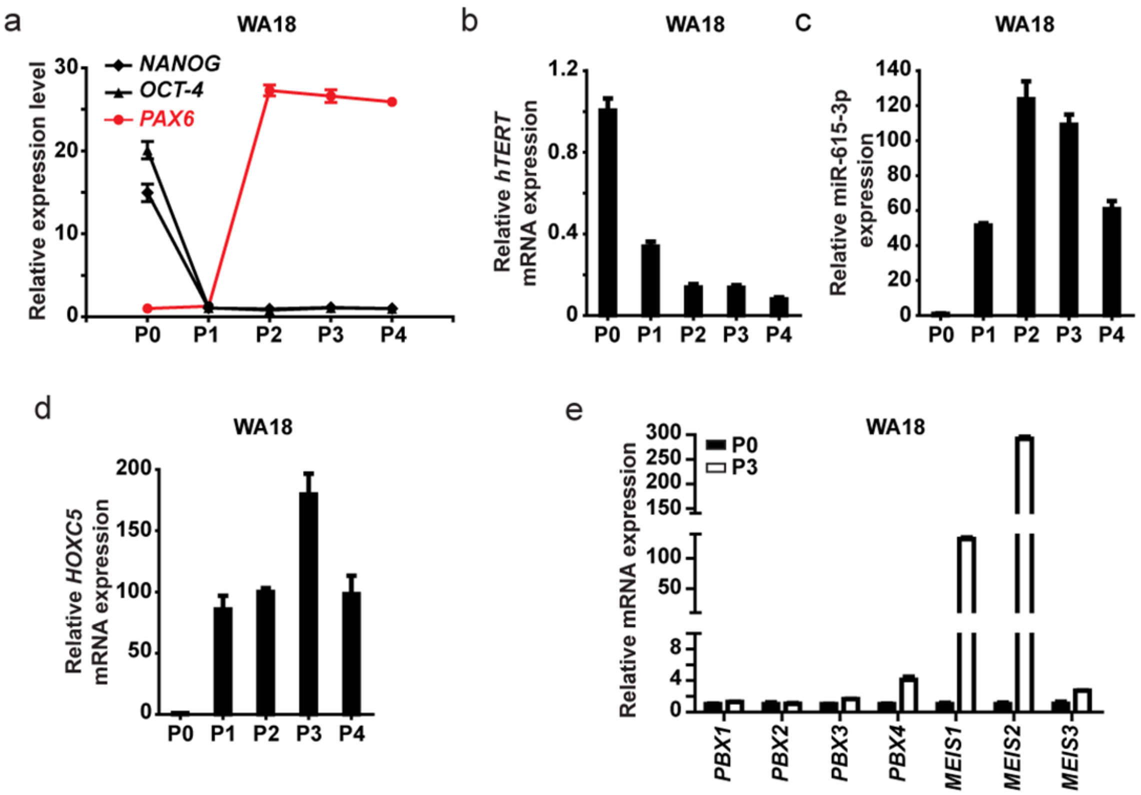

Supplementary Figure 6: Expression of *hTERT*, miR-615-3p, *HOXC5, PBX1-4*, and *MEIS1-3* in WA18 human ES cells upon neural induction.

a. The expression of pluripotency genes (*NANOG* and *OCT-4*) and neurodevelopmental gene *PAX6* during neural differentiation (passage 0 to 4) in WA18 ES cells were quantified by real-time RT-PCR.

b. c. and d. The expression of *hTERT* mRNA, miR-615-3p and *HOXC5* mRNA during neural differentiation (passage 0 to 4) in WA18 cells was quantified by real-time RT-PCR as indicated.

e. The expression of *PBX1-4* and *MEIS1-3* in P0 and P3 of WA18 neural differentiation was quantified by real-time RT-PCR as indicated.

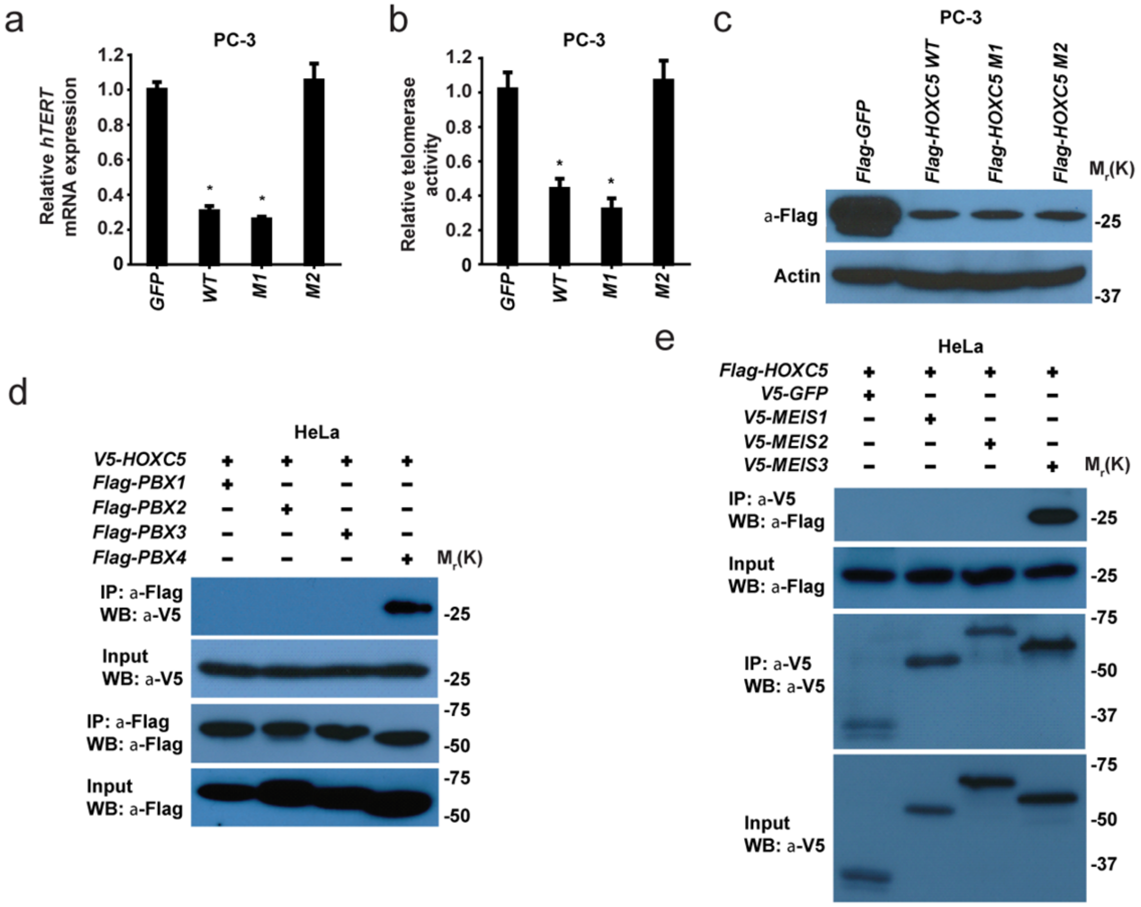

Supplementary Figure 7: HoxC5 recruits Pbx4 and Meis3 to suppress *hTERT* expression.

a. Relative *hTERT* mRNA expression in PC-3 cells transduced with lentivirus overexpressing Flag-tagged GFP, wild-type (WT), or mutant (M1 or M2) *HOXC5*. Significance was determined by t-test. * $P \leq 0.05$.

b. Relative telomerase activity in PC-3 cells transduced with lentivirus overexpressing Flag-tagged GFP, wild-type (WT), or mutant (M1 or M2) *HOXC5*. Significance was determined by t-test. * $P \leq 0.05$.

c. Western blotting shows the expression of Flag-tagged GFP, wild-type (WT) and mutant (M1 or M2) HoxC5 expressed in PC-3 cells.

d. Immunoprecipitation of Flag-tagged Pbx4, but not Flag-tagged Pbx1, 2 or 3 resulted in specific co-immunoprecipitation of V5-tagged HoxC5 in HeLa cells.

e. Immunoprecipitation of V5-tagged Meis3, but not V5-tagged Meis1 or 2 resulted in specific co-immunoprecipitation of Flag-tagged HoxC5 in HeLa cells.

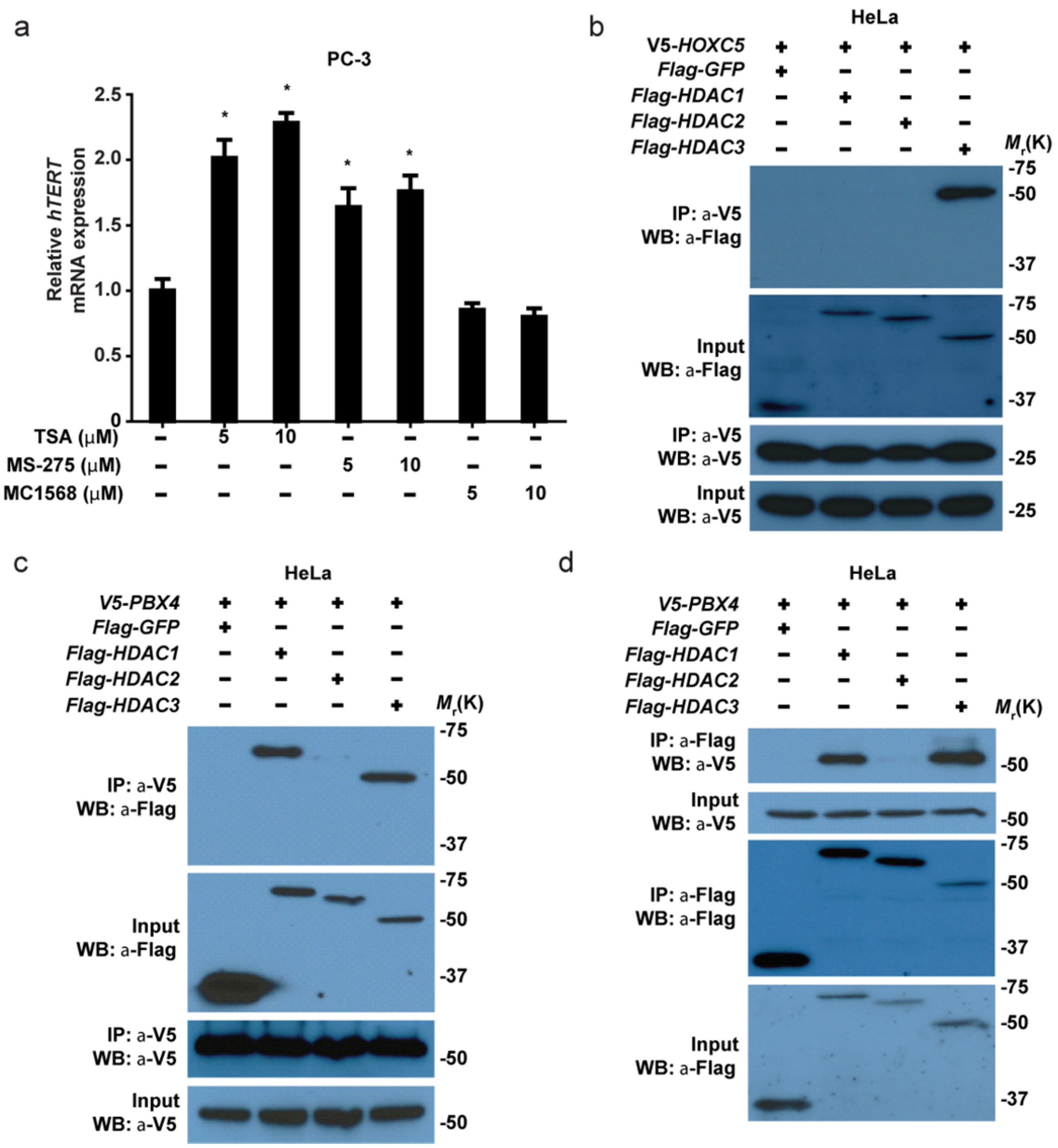

Supplementary Figure 8: HDAC1 and 3 containing co-repressor complex is necessary for HoxC5-Pbx4 mediated suppression of *hTERT* expression.

a. The expression of *hTERT* mRNA in PC-3 cells treated with HDAC inhibitors TSA, MS-275 or MC1568 as quantified by real-time RT-PCR. Significance was determined by t-test. * $P \leq 0.05$.

b. Immunoprecipitation of V5-tagged HoxC5 resulted in co-immunoprecipitation of Flag-tagged HDAC3, but not Flag-tagged HDAC1 or 2 in HeLa cells.

c. Immunoprecipitation of V5-tagged Pbx4 resulted in specific co-immunoprecipitation of Flag-tagged HDAC1 and 3, but not HDAC2 in HeLa cells.

d. Immunoprecipitation of Flag-tagged HDAC1 and 3, but not HDAC2, resulted in efficient co-immunoprecipitation of V5-tagged Pbx4 in HeLa cells.

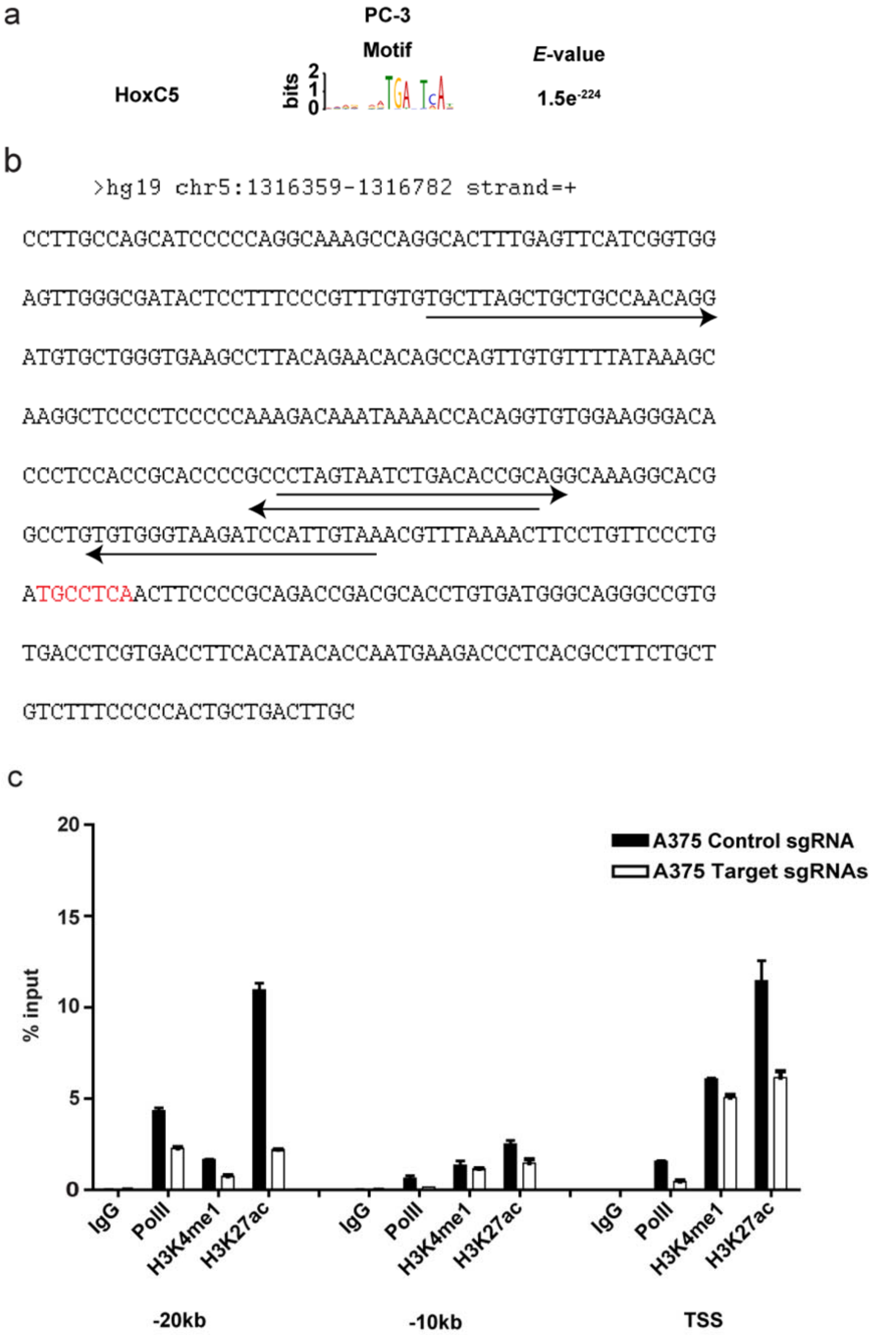

Supplementary Figure 9: Identification of HoxC5 DNA binding motif in *hTERT* upstream enhancer region.

a. The consensus DNA binding motif of HoxC5 was detected using MEME on the endogenous HoxC5 binding sites in PC-3 cells.

b. The putative binding motif in the *hTERT* -20 kb upstream HoxC5 binding region is highlighted in red. The four sgRNAs used in CRISPRi experiment are indicated by arrows.

c. ChIP was performed against RNA polymerase II (Pol2), H3K4me1, and H3K27ac in A375 cells co-expressing dCas9-KRAB with control sgRNA or sgRNAs targeting the HoxC5 binding region at upstream *hTERT* enhancer followed by qPCR with primers specific for *hTERT* TSS, -10kb and -20kb upstream regions as indicated.

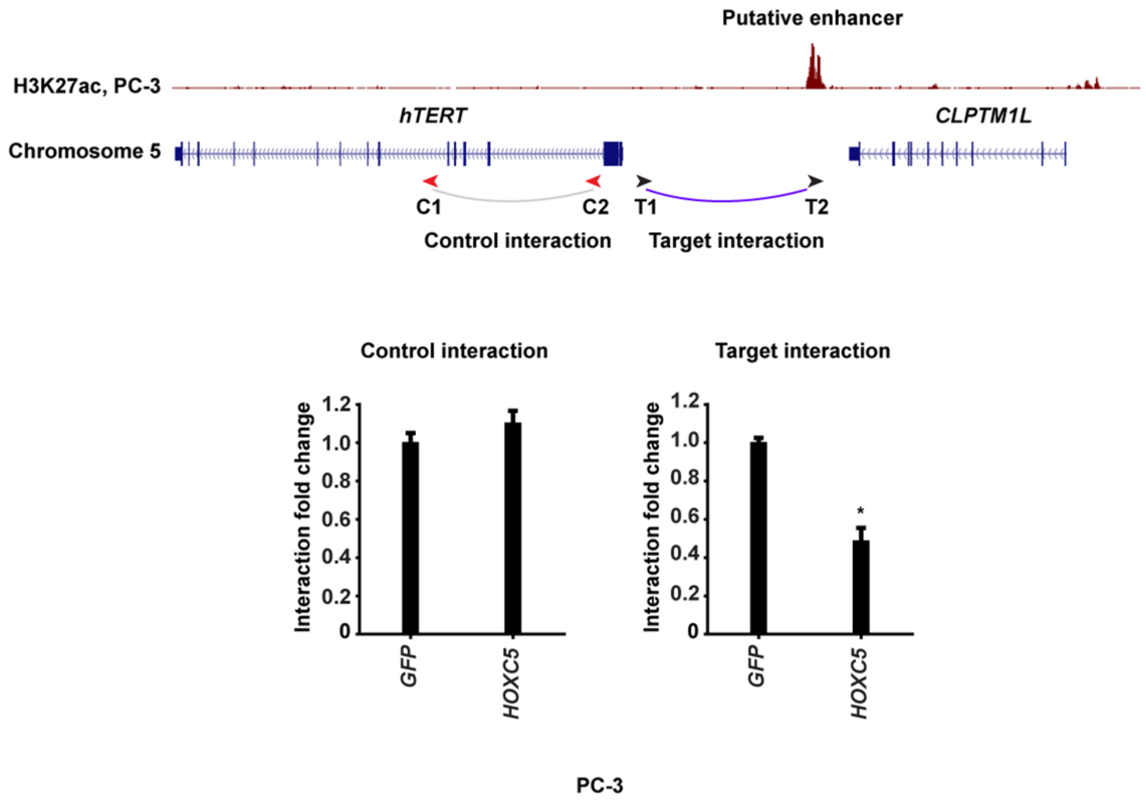

Supplementary Figure 10: Overexpression of *HOXC5* in PC-3 cells disrupts the long-range interaction between *hTERT* promoter and distal enhancer. Control interaction: Based on the 4C-seq data, we selected a region in the *hTERT* coding region with background levels of interaction, with a similar genomic distance from the *hTERT* bait region to serve as a control interaction. Target interaction: long-range interaction between *hTERT* promoter and putative enhancer. Significance was determined by t-test. * $P \leq 0.05$.

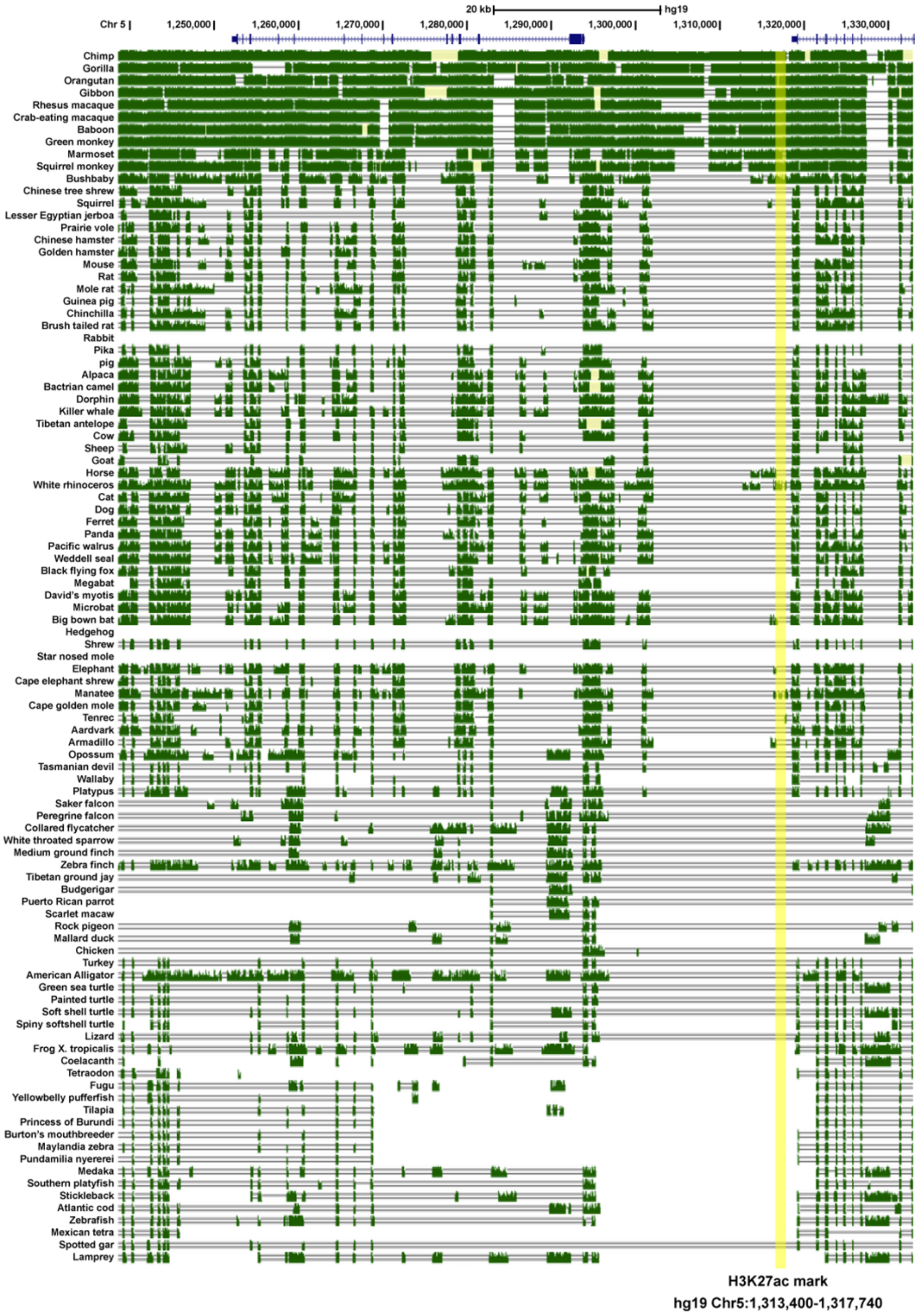

Supplementary Figure 11: The conservation of *hTERT* upstream enhancer region in 100 vertebrate species. Pairwise alignments of each species to the human genome are displayed as histogram to indicate conservation score. The upstream enhancer region is highlighted in yellow.

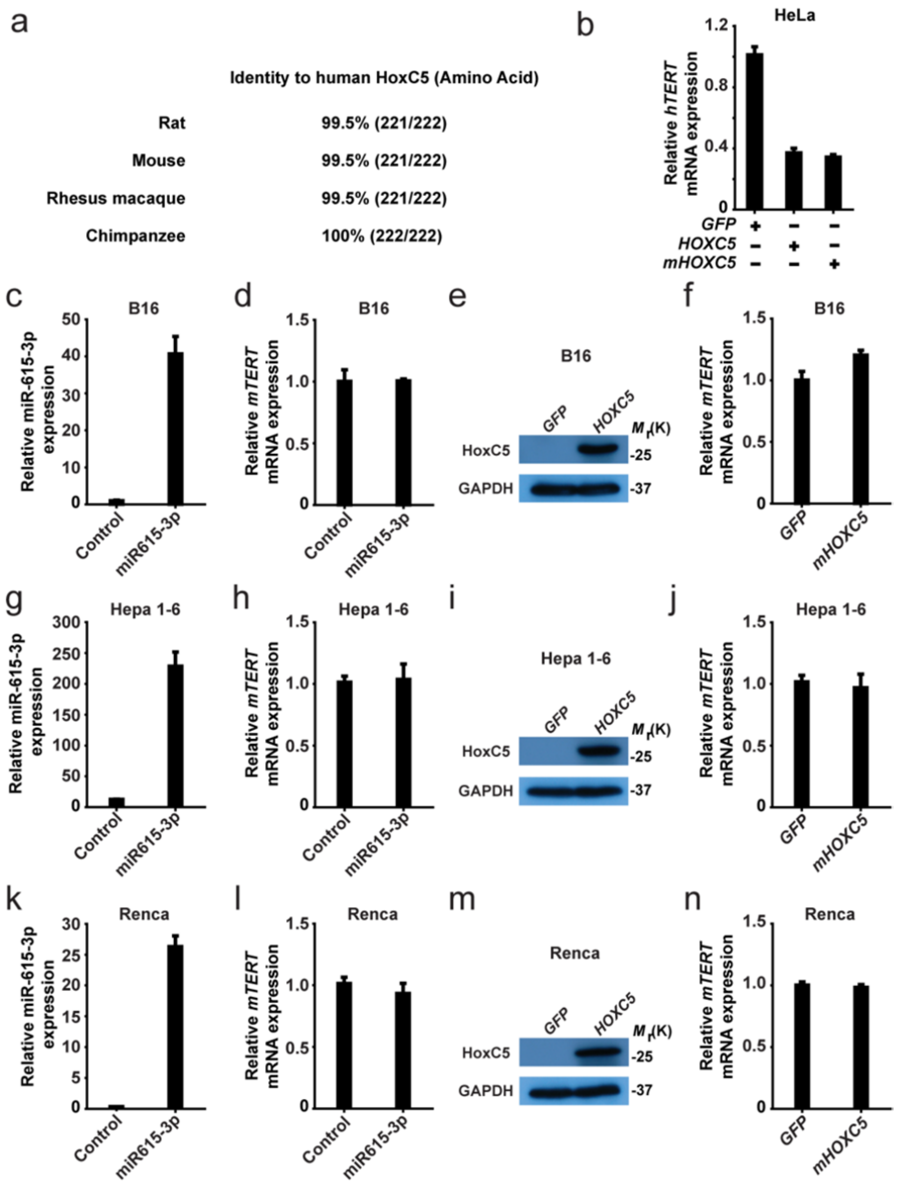

Supplementary Figure 12: Distinct function of *HOXC5* in regulation of *TERT* expression in human and mouse cells.

a. The identity of HoxC5 protein from different species in comparison to human HoxC5 protein was indicated.

b. Relative *hTERT* mRNA expression in HeLa transduced with lentivirus overexpressing Flag-tagged human or mouse *HOXC5*.

c. g. and k. Relative miR-615-3p expression in B16, Hepa 1-6 and Renca mouse cells transduced with control lentivirus or lentivirus overexpressing miR-615-3p.

d. h. and l. Relative *mTERT* mRNA expression in B16, Hepa 1-6 and Renca mouse cells transduced with control lentivirus or lentivirus overexpressing miR-615-3p.

e. i. and m. Western blotting showing overexpression of mHoxC5 in B16, Hepa 1-6 and Renca mouse cells transduced with control GFP lentivirus or lentivirus overexpressing *mHOXC5*.

f. j. and n. Relative *mTERT* mRNA expression in B16, Hepa 1-6 and Renca mouse cells transduced with control lentivirus or lentivirus overexpressing *mHOXC5*.

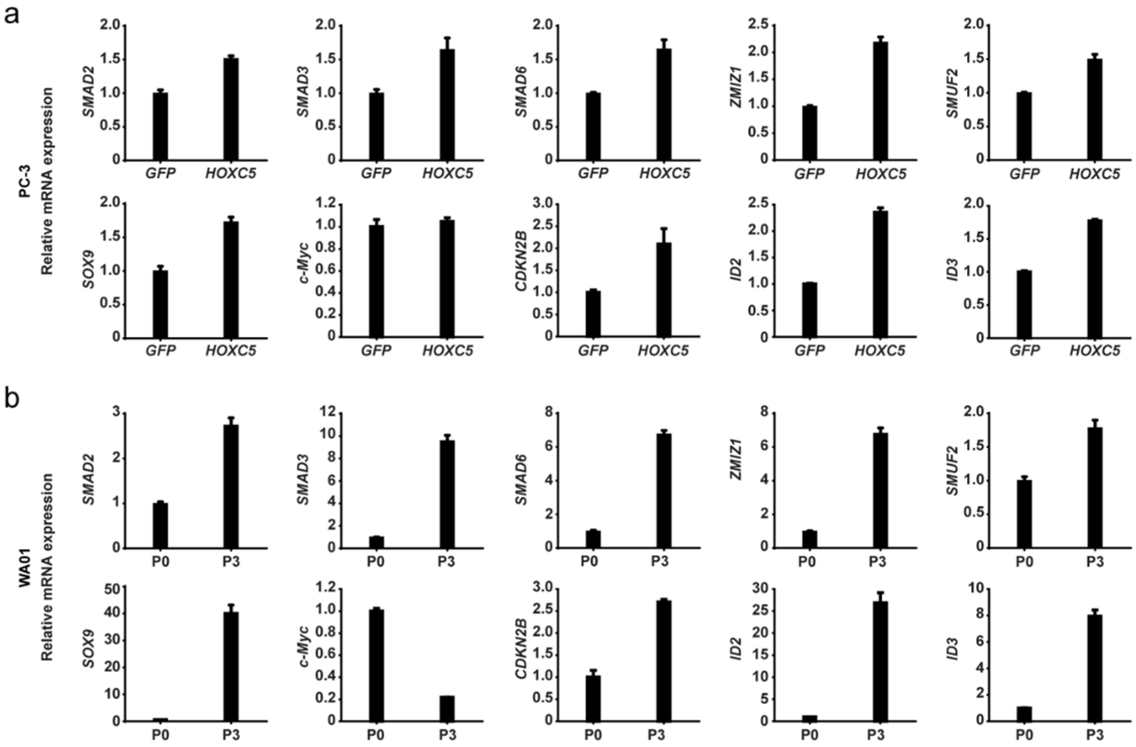

Supplementary Figure 13: The expression of TGF-β target genes

a. The expression of TGF-β target genes in PC-3 cells overexpressing control *GFP* or *HOXC5* was quantified using real-time RT-PCR.

b. The expression of TGF-β target genes in human WA01 ES cells before or after neural induction was quantified using real-time RT-PCR.

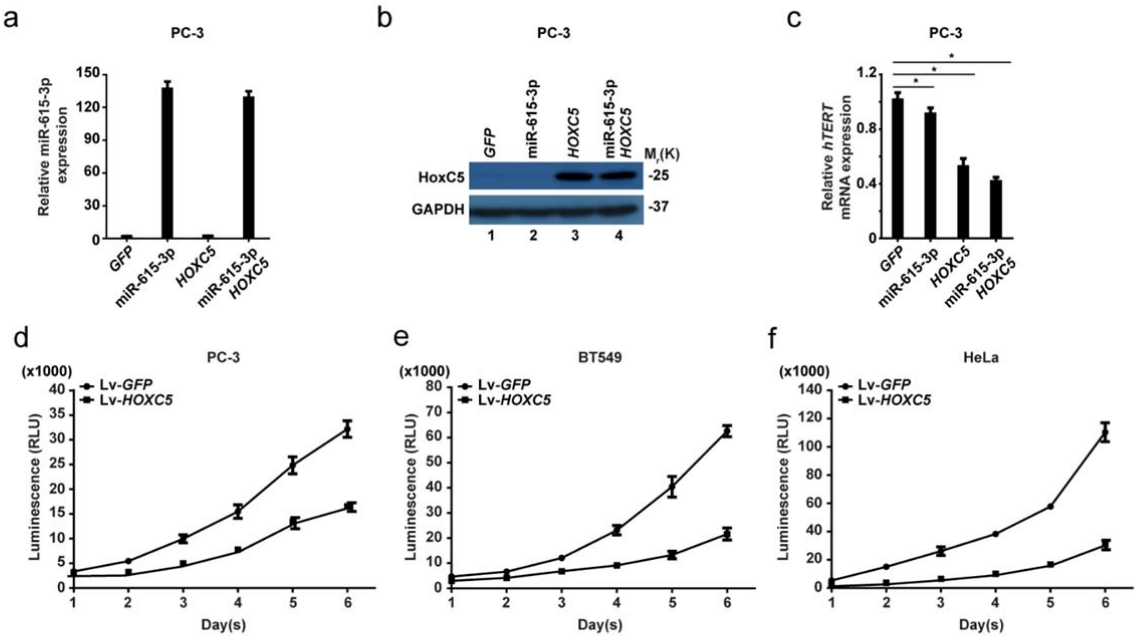

Supplementary Figure 14: Overexpression of *HOXC5* inhibits cancer cell proliferation.
a. Relative expression of miR-615-3p in PC-3 cells that were transduced with lentivirus expressing *GFP*, *mir-615-3p*, *Flag-HOXC5* or both *Flag-HOXC5* and *mir-615-3p* as quantified using real-time RT-PCR.
b. The expression of Flag-tagged HoxC5 in PC-3 cells that were transduced with lentivirus expressing *GFP*, *mir-615-3p*, *Flag-HOXC5* or both *Flag-HOXC5* and *mir-615-3p* as shown by Western blotting.
c. Relative expression of *hTERT* mRNA in PC-3 cells that were transduced with lentivirus expressing *GFP*, *mir-615-3p*, *Flag-HOXC5* or both *Flag-HOXC5* and *mir-615-3p* as quantified using real-time RT-PCR. Significance was determined by t-test. * $P \leq 0.05$.
d. e. and f. Overexpression of HOXC5, but not GFP inhibits PC-3, BT549 and HeLa cancer cells' proliferation as shown by CellTiter-Glo luminescent cell viability assay.

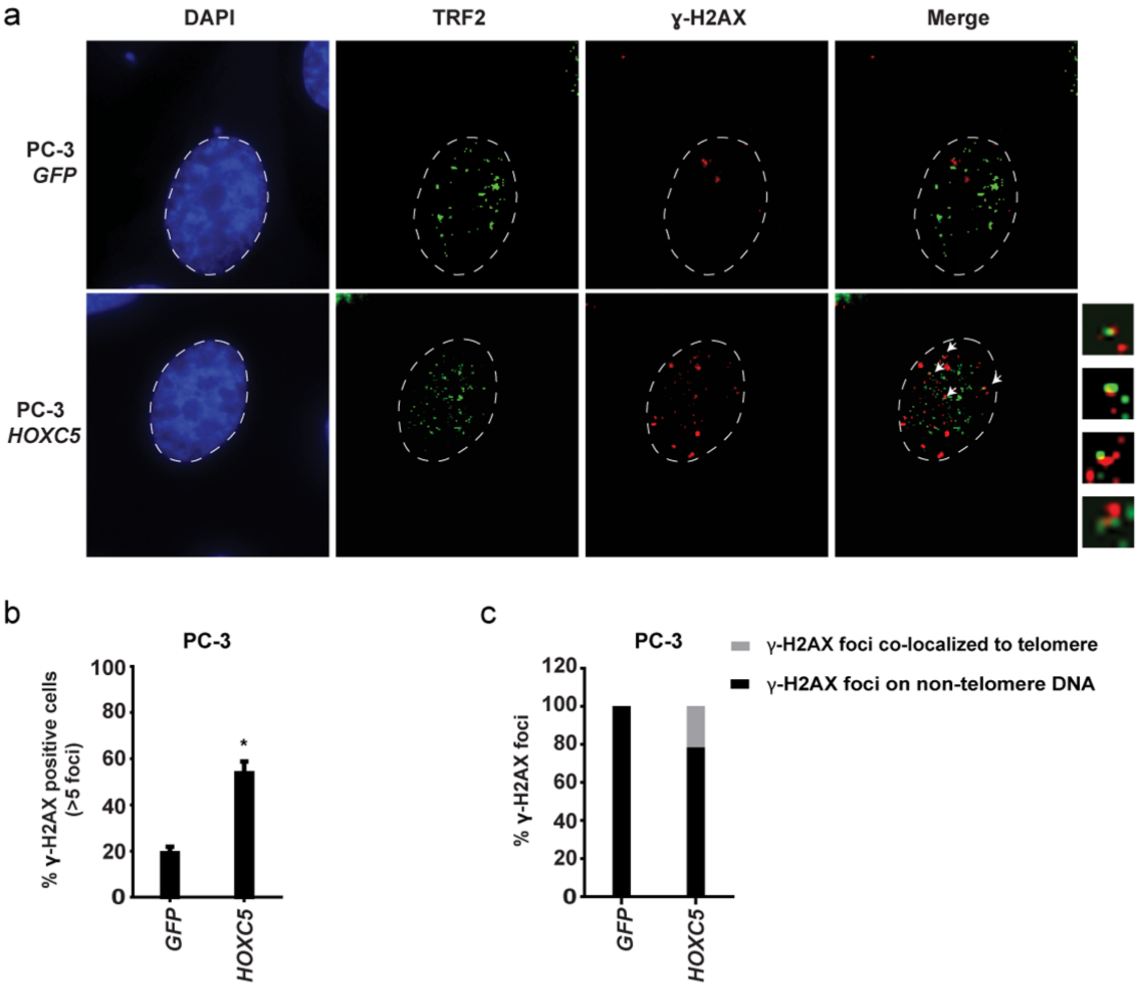

Supplementary Figure 15: Detection of telomere dysfunction-induced foci (TIF) in PC-3 cells overexpressing *HOXC5*.

a. Overexpression of *HOXC5*, but not *GFP* in PC-3 cells induced telomeric localization of γ-H2AX. Cells were processed for DNA staining (DAPI, blue) as well as double immunostaining of TRF2 (green) and γ-H2AX (red). Colocalization of TRF2 and γ-H2AX is indicated by arrow and showed in enlarged pictures.

b. Comparison of γ-H2AX-positive cells in PC-3 cells overexpressing *GFP* or *HOXC5*. Bars represent the mean percentage+/-SD. Significance was determined by t-test. * $P \leq 0.05$.

c. Quantification of γ-H2AX foci detected in a. Signal of γ-H2AX foci overlapping telomeres (gray) and those not overlapping (black) are shown separately. A minimum of 100 cells were analyzed for each cell line.

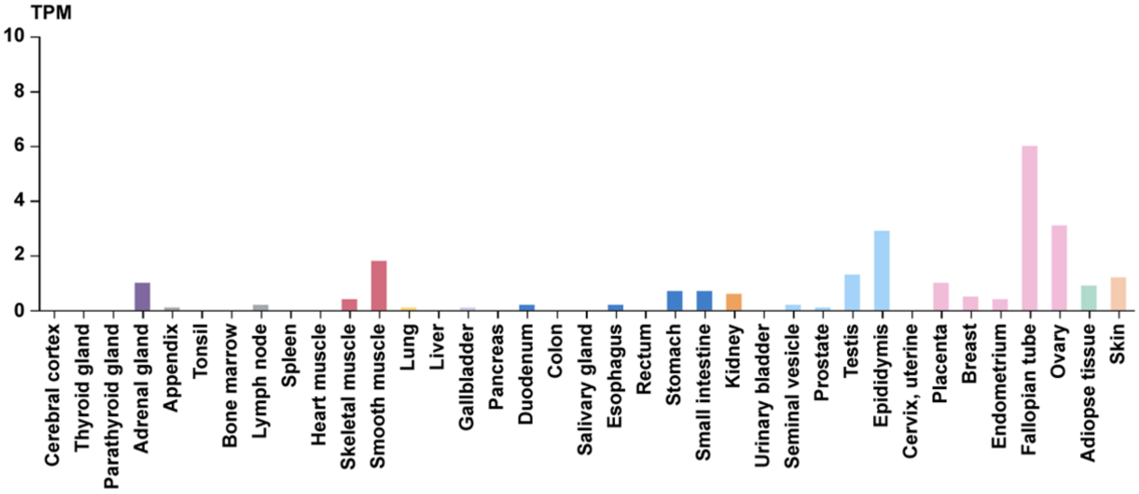

http://www.proteinatlas.org/ENSG00000172789-HOXC5/tissue

Supplementary Figure 16: Expression of *HOXC5* mRNA in human tissues.

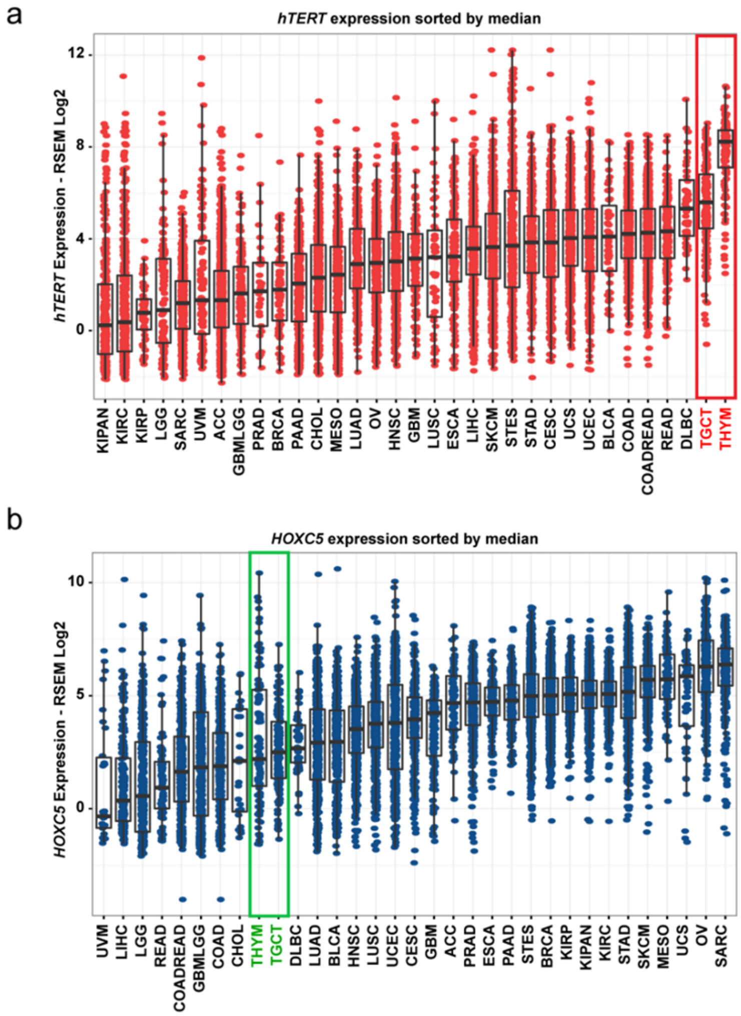

Supplementary Figure 17: Expression of *hTERT* and *HOXC5* (Log2 RSEM) in 33 different TCGA cancer types.

a. Boxplot of *hTERT* expression in 33 TCGA cancer types, sorted by median *hTERT* expression. TGCT and THYM (highlighted in red box) have the highest median expression (5.58 RSEM log2 units for TGCT and 8.23 RSEM log2 units for THYM).

b. Boxplot of *HOXC5* expression in 33 TCGA cancer types, sorted by median *HOXC5* expression. TGCT and THYM (highlighted in green box) have low median expression (2.49 for TGCT and 2.19 for THYM).

KIPAN: Pan-Kidney Cohort; KIRC: Kidney Renal Clear Cell Carcinoma; KIRP: Kidney Renal Papillary Carcinoma; LGG: Brain Lower Grade Glioma; SARC: Sarcoma; UVM: Uveal Melanoma; ACC: Adrenocortical Carcinoma; GBMLGG: Glioma; PRAD: Prostate Adenocarcinoma; BRCA: Breast invasive carcinoma; PAAD: Pancreatic Adenocarcinoma; CHOL: Cholangiocarcinoma; MESO: Mesothelioma; LUAD: Lung Adenocarcinoma; OV: Ovarian Serous Cystadenocarcinoma; HNSC: Head and Neck Squamous Cell Carcinoma; GBM: Glioblastoma Multiforme; LUSC: Lung Squamous Cell Carcinoma; ESCA: Esophageal Carcinoma; LIHC: Liver Hepatocellular Carcinoma; SKCM: Skin Cutaneous Melanoma; STES: Stomach and Esophageal Carcinoma; STAD: Stomach Adenocarcinoma; CESC: Cervical and Endocervical Cancers; UCS: Uterine Carcinosarcoma; UCEC: Uterine Corpus Endometrial Carcinoma; BLCA: Bladder Urothelial Carcinoma; COAD: Colon Adenocarcinoma; COADREAD: Colorectal Adenocarcinoma; READ: Rectum Adenocarcinoma; DLBC: Lymphoid Neoplasm Diffuse Large B-Cell Lymphoma; TGCT: Testicular Germ Cell Tumor; THYM: Thymoma.

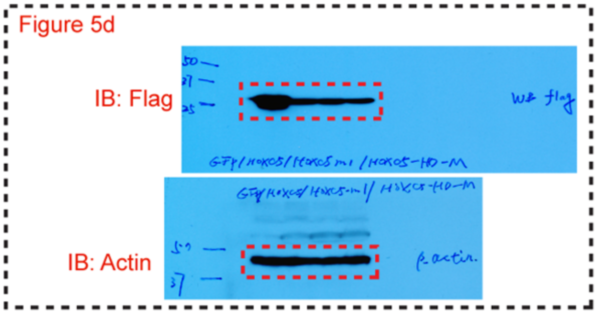
Figure 5d
IB: Flag
IB: Actin
50
37
25

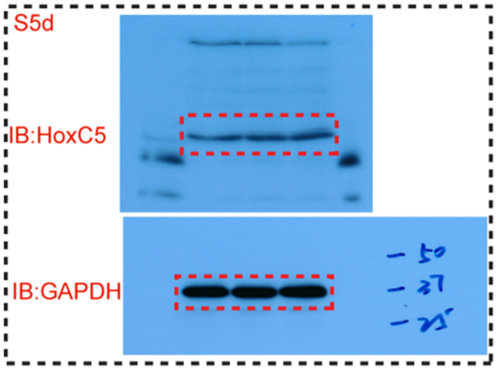
S5d
IB:HoxC5
IB:GAPDH
50
37
25

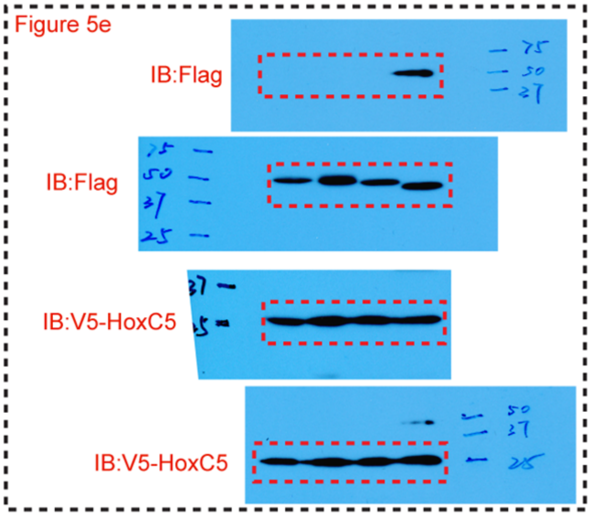
Figure 5e
IB:Flag
IB:Flag
IB:V5-HoxC5
IB:V5-HoxC5
75
50
37
25

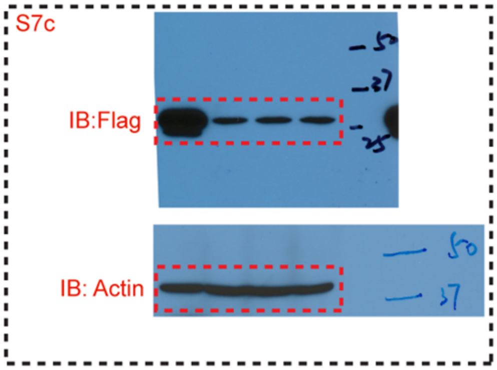
S7c
IB:Flag
IB: Actin
50
37
25

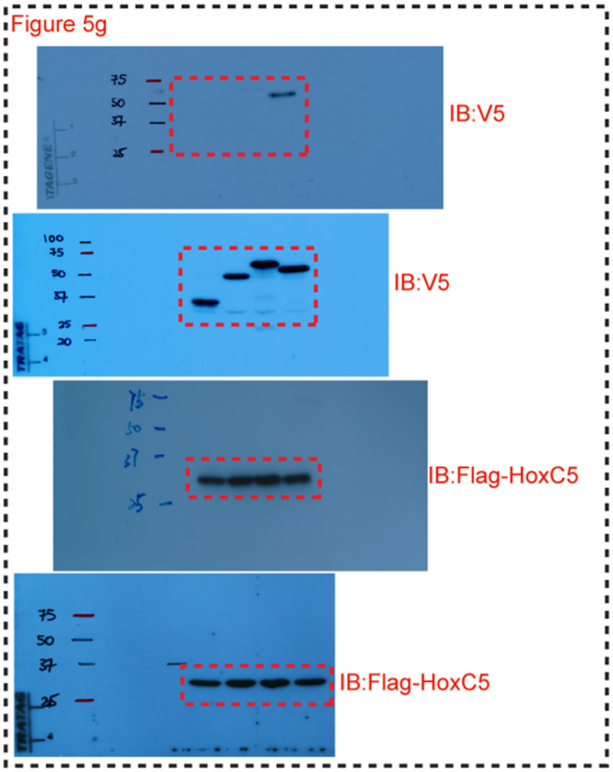
Figure 5g
IB:V5
IB:V5
IB:Flag-HoxC5
IB:Flag-HoxC5
100
75
50
37
25
20

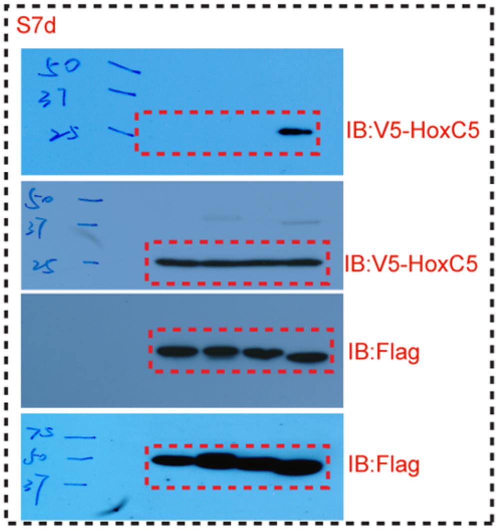
S7d
IB:V5-HoxC5
IB:V5-HoxC5
IB:Flag
IB:Flag
75
50
37
25

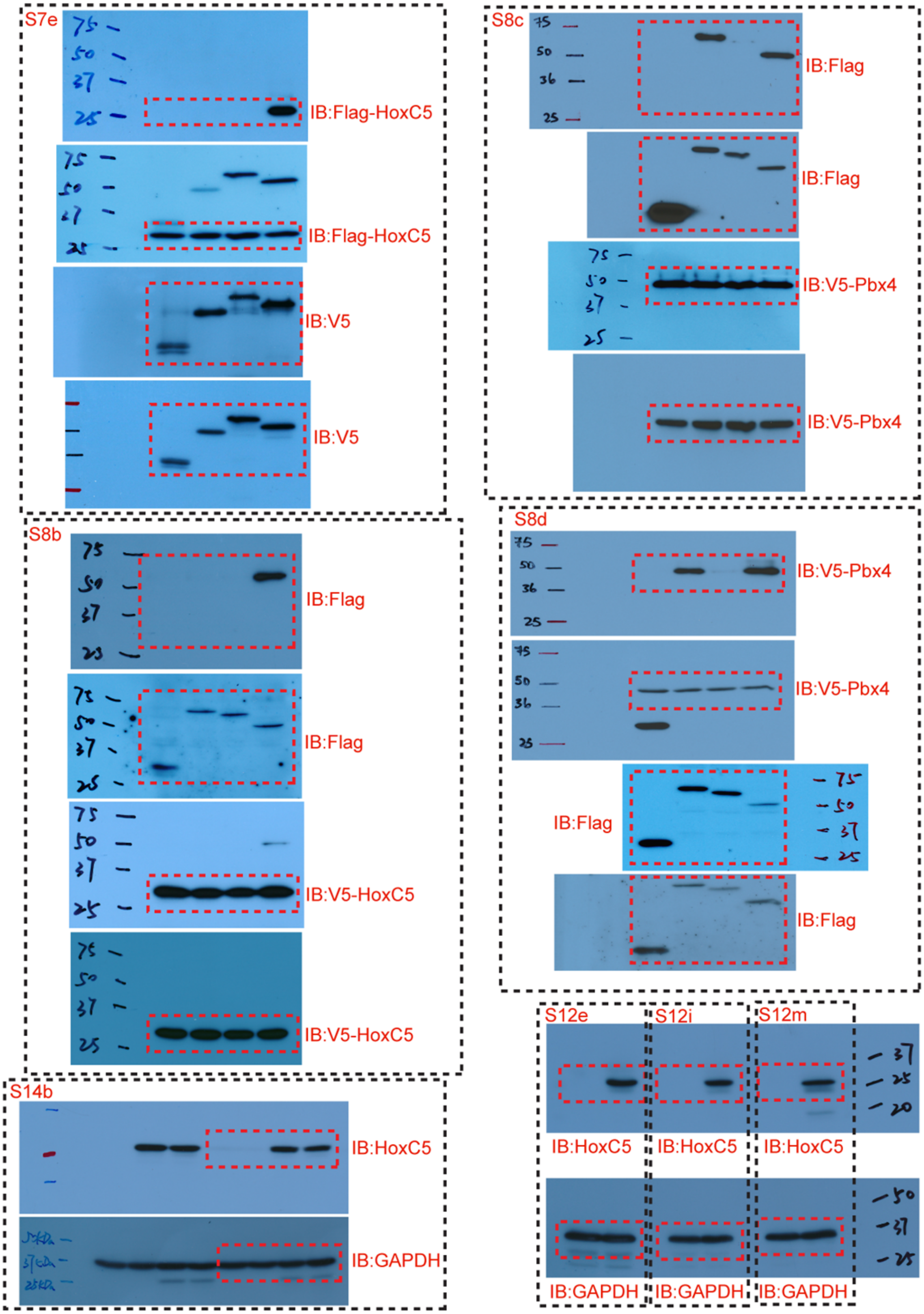

Supplementary Figure 18: The uncropped western blot scans.

Supplementary Tables

Supplementary Table 1: The potential targets of miR-615-3p predicted using the microT-CDS algorithm.

Ensembl Gene Id	miRNA name	miTG score	Ensembl Gene Id		
ENSG00000204052 (LRRC73)	hsa-miR-615-3p	0.996363852	LRRC73	Also predicted by Targetscan	
ENSG00000149485 (FADS1)	hsa-miR-615-3p	0.991069849	FADS1	Also predicted by Targetscan	
ENSG00000120899 (PTK2B)	hsa-miR-615-3p	0.967646448	PTK2B	Also predicted by Targetscan	
ENSG00000163701 (IL17RE)	hsa-miR-615-3p	0.960641477	IL17RE	Also predicted by Targetscan	
NSG00000204422 (XXbac-BPG32J3.20)	hsa-miR-615-3p	0.950346894	XXbac-BPG32J3.20	Also predicted by Targetscan	
ENSG00000141665 (FBXO15)	hsa-miR-615-3p	0.948559866	FBXO15	Also predicted by Targetscan	
ENSG00000204427 (ABHD16A)	hsa-miR-615-3p	0.948532097	ABHD16A	Also predicted by Targetscan	
ENSG00000186104 (CYP2R1)	hsa-miR-615-3p	0.942345735	CYP2R1	Also predicted by Targetscan	
ENSG00000068305 (MEF2A)	hsa-miR-615-3p	0.940511833	MEF2A	Also predicted by Targetscan	
ENSG00000099330 (OCEL1)	hsa-miR-615-3p	0.932861434	OCEL1	Also predicted by Targetscan	
ENSG00000155367 (PPM1J)	hsa-miR-615-3p	0.918537497	PPM1J	Also predicted by Targetscan	
ENSG00000168488 (ATXN2L)	hsa-miR-615-3p	0.905164923	ATXN2L	Also predicted by Targetscan	
ENSG00000010322 (NISCH)	hsa-miR-615-3p	0.903871908	NISCH	Also predicted by Targetscan	
ENSG00000162650 (ATXN7L2)	hsa-miR-615-3p	0.900254306	ATXN7L2	Also predicted by Targetscan	
ENSG00000196683 (TOMM7)	hsa-miR-615-3p	0.896126765	TOMM7	Also predicted by Targetscan	
ENSG00000114857 (NKTR)	hsa-miR-615-3p	0.891295434	NKTR	Also predicted by Targetscan	
ENSG00000264058 (KRT222)	hsa-miR-615-3p	0.87832565	KRT222	Also predicted by Targetscan	
ENSG00000125304 (TM9SF2)	hsa-miR-615-3p	0.870107894	TM9SF2	Also predicted by Targetscan	
ENSG00000130950 (NUTM2F)	hsa-miR-615-3p	0.868009628	NUTM2F	Also predicted by Targetscan	
ENSG00000136014 (USP44)	hsa-miR-615-3p	0.866328459	USP44	Also predicted by Targetscan	
ENSG00000166436 (TRIM66)	hsa-miR-615-3p	0.860439158	TRIM66	Also predicted by Targetscan	
ENSG00000177627 (C12orf54)	hsa-miR-615-3p	0.859569117	C12orf54	Also predicted by Targetscan	
ENSG00000213024 (NUP62)	hsa-miR-615-3p	0.853266206	NUP62	Also predicted by Targetscan	
ENSG00000172878 (METAP1D)	hsa-miR-615-3p	0.845137038	METAP1D	Also predicted by Targetscan	
ENSG00000090006 (LTBP4)	hsa-miR-615-3p	0.843511571	LTBP4	Also predicted by Targetscan	
ENSG00000130208 (APOC1)	hsa-miR-615-3p	0.836413839	APOC1	Also predicted by Targetscan	
ENSG00000154146 (NRGN)	hsa-miR-615-3p	0.835987146	NRGN	Also predicted by Targetscan	
ENSG00000134014 (ELP3)	hsa-miR-615-3p	0.8359661	ELP3	Also predicted by Targetscan	
ENSG00000184368 (MAP7D2)	hsa-miR-615-3p	0.835571226	MAP7D2	Also predicted by Targetscan	
ENSG00000108671 (PSMD11)	hsa-miR-615-3p	0.827397667	PSMD11	Also predicted by Targetscan	
ENSG00000072778 (ACADVL)	hsa-miR-615-3p	0.823677075	ACADVL	Also predicted by Targetscan	
ENSG00000163956 (LRPAP1)	hsa-miR-615-3p	0.823040269	LRPAP1	Also predicted by Targetscan	
ENSG00000106012 (IQCE)	hsa-miR-615-3p	0.81064738	IQCE	Also predicted by Targetscan	
ENSG00000219200 (RNASEK)	hsa-miR-615-3p	0.808783768	RNASEK	Also predicted by Targetscan	Experimentally verified
ENSG00000173171 (MTX1)	hsa-miR-615-3p	0.80098187	MTX1	Also predicted by Targetscan	
ENSG00000105737 (GRIK5)	hsa-miR-615-3p	0.798594078	GRIK5	Also predicted by Targetscan	
ENSG00000013503 (POLR3B)	hsa-miR-615-3p	0.791981561	POLR3B	Also predicted by Targetscan	
ENSG00000186868 (MAPT)	hsa-miR-615-3p	0.787900988	MAPT	Also predicted by Targetscan	
ENSG00000175203 (DCTN2)	hsa-miR-615-3p	0.786717445	DCTN2	Also predicted by Targetscan	
ENSG00000179057 (IGSF22)	hsa-miR-615-3p	0.783672468	IGSF22	Also predicted by Targetscan	
ENSG00000188171 (ZNF626)	hsa-miR-615-3p	0.782953458	ZNF626	Also predicted by Targetscan	
ENSG00000167858 (TEKT1)	hsa-miR-615-3p	0.773304055	TEKT1	Also predicted by Targetscan	
ENSG00000135441 (BLOC1S1)	hsa-miR-615-3p	0.771625171	BLOC1S1	Also predicted by Targetscan	
ENSG00000179133 (C10orf67)	hsa-miR-615-3p	0.769947324	C10orf67	Also predicted by Targetscan	
ENSG00000139567 (ACVRL1)	hsa-miR-615-3p	0.766832637	ACVRL1	Also predicted by Targetscan	
ENSG00000132155 (RAF1)	hsa-miR-615-3p	0.766538016	RAF1	Also predicted by Targetscan	
ENSG00000163803 (PLB1)	hsa-miR-615-3p	0.764041997	PLB1	Also predicted by Targetscan	
ENSG00000102312 (PORCN)	hsa-miR-615-3p	0.763990626	PORCN	Also predicted by Targetscan	
ENSG00000143164 (DCAF6)	hsa-miR-615-3p	0.757564443	DCAF6	Also predicted by Targetscan	
ENSG00000187486 (KCNJ11)	hsa-miR-615-3p	0.754144971	KCNJ11	Also predicted by Targetscan	
ENSG00000168487 (BMP1)	hsa-miR-615-3p	0.752624187	BMP1	Also predicted by Targetscan	
ENSG00000182504 (CEP97)	hsa-miR-615-3p	0.749143701	CEP97	Also predicted by Targetscan	
ENSG00000130055 (GDPD2)	hsa-miR-615-3p	0.747628184	GDPD2	Also predicted by Targetscan	
ENSG00000072071 (LPHN1)	hsa-miR-615-3p	0.734297485	LPHN1	Also predicted by Targetscan	
ENSG00000101577 (LPIN2)	hsa-miR-615-3p	0.72952031	LPIN2	Also predicted by Targetscan	
ENSG00000185033 (SEMA4B)	hsa-miR-615-3p	0.719097546	SEMA4B	Also predicted by Targetscan	
ENSG00000170310 (STX8)	hsa-miR-615-3p	0.713477468	STX8		
ENSG00000187147 (RNF220)	hsa-miR-615-3p	0.704806453	RNF220		
ENSG00000115207 (GTF3C2)	hsa-miR-615-3p	0.702925014	GTF3C2		

Supplementary Table 2: Primer sequences for 3C experiments.

Name	Sequence
Control interaction primer C1	CGGTCGTAGGGTCTGATGTG
Control interaction primer C2	GTCCTCCTGTCTCCATCGTC
Target interaction T1	GTTATTAAGCCAGGCTCAGACT
Target interaction T2	CCGCCCTAGTAATCTGACAC
loading control RP	AGTCCCGCACGCTCATCTT
loading control FP	ACCAAGAAGTTCATCTCCCTGG
